# Plasma Amyloid-β dynamics in late-life major depression: a longitudinal study

**DOI:** 10.1038/s41398-022-02077-8

**Published:** 2022-07-28

**Authors:** Nunzio Pomara, Davide Bruno, Chelsea Reichert Plaska, Jaime Ramos-Cejudo, Ricardo S. Osorio, Anilkumar Pillai, Bruno P. Imbimbo, Henrik Zetterberg, Kaj Blennow

**Affiliations:** 1 Nathan Kline Institute, Orangeburg, NY USA; 2 Department of Psychiatry and Pathology, New York University-Grossman School of Medicine, New York, NY USA; 3 School of Psychology, Liverpool John Moores University, Liverpool, UK; 4 Department of Psychiatry, New York University-Grossman School of Medicine, New York, NY USA; 5 Pathophysiology of Neuropsychiatric Disorders Program, Faillace Department of Psychiatry and Behavioral Sciences, McGovern Medical School, The University of Texas Health Science Center at Houston (UTHealth), Houston, TX USA; 6 Research and Development, Charlie Norwood VA Medical Center, Augusta, GA USA; 7 Department of Psychiatry and Health Behavior, Medical College of Georgia, Augusta University, Augusta, GA USA; 8 Research & Development, Chiesi Farmaceutici, Parma, Italy; 9 Department of Neurodegenerative Disease, UCL Institute of Neurology, London, UK; 10 UK Dementia Research Institute at UCL, London, UK; 11 Department of Psychiatry and Neurochemistry, Institute of Neuroscience and Physiology, the Sahlgrenska Academy at the University of Gothenburg, Mölndal, Sweden; 12 Clinical Neurochemistry Laboratory, Sahlgrenska University Hospital, Mölndal, Sweden; 13 Hong Kong Center for Neurodegenerative Diseases, Clear Water Bay, Hong Kong, China

**Keywords:** Predictive markers, Depression

## Abstract

Depressed individuals are twice as likely to develop Alzheimer’s disease (AD) as compared to controls. Brain amyloid-β (Aβ) deposition is believed to have a major role in AD pathogenesis but studies also suggest associations of Aβ dynamics and depression. The aim of this study was to test if plasma Aβ levels are longitudinally associated to late-life depression. We measured plasma levels of amyloid-β_1-40_ (Aβ40) and amyloid-β_1-42_ (Aβ42) peptides longitudinally for three consecutive years in 48 cognitively intact elderly subjects with late-life major depressive disorder (LLMD) and 45 age-matched cognitively healthy controls. We found that the Aβ42/Aβ40 plasma ratio was significantly and steadily lower in depressed subjects compared to controls (*p* < 0.001). At screening, Aβ42/Aβ40 plasma did not correlate with depression severity (as measured with Hamilton Depression Scale) or cognitive performance (as measured with Mini-Mental State Examination) but was associated to depression severity at 3 years after adjustment for age, education, cognitive performance, and antidepressants use. This study showed that reduced plasma Aβ42/Aβ40 ratio is consistently associated with LLMD diagnosis and that increased severity of depression at baseline predicted low Aβ42/Aβ40 ratio at 3 years. Future studies are needed to confirm these findings and examine if the consistently lower plasma Aβ42/Aβ40 ratio in LLMD reflects increased brain amyloid deposition, as observed in AD subjects, and an increased risk for progressive cognitive decline and AD.

## Introduction

Major depressive disorder (MDD) is a well-established risk factor for Alzheimer’s disease (AD) [1, 2]. Depression is also a part of the clinical syndrome in the early stages of AD and can be a prodromal manifestation [3, 4]. A meta-analysis of studies of depression and dementia [5] showed that depressed individuals are nearly twice as likely to develop dementia as compared to controls [6]. A 7-year longitudinal study [7] showed that for each point increase in the 10-item Center for Epidemiologic Studies Depression Scale (CES-D), risk of developing AD increased by an average of 19%. Lifetime depression has been associated with increases in AD-related brain pathology, including elevated density of neuritic plaques and neurofibrillary tangles [8, 9]. However, not all studies have found a link between depression and AD [10–12], and depression may be part of the dementia prodrome thus highlighting the complexity of the relationship between these two conditions.

In a bid to elucidate the relationship between depression and AD, several reports, including from our group [13], have suggested that disturbances in amyloid-β (Aβ) production or aggregation status may play a role in the disease onset, alone or in combination with other factors (e.g., inflammation). Aβ deposition in the brain is a hallmark of AD, but abnormal Aβ levels have also been observed in individuals with MDD, both in the cerebrospinal fluid [13], and in the brain [14, 15]. In AD, numerous studies have shown a reduction in both the plasma and CSF Aβ42/Aβ40 ratio which has been associated with increases in amyloid PET uptake [16, 17]. Studies of PET amyloid scans in MDD have provided conflicting results with some reports of increased amyloid build up [14, 18–21], or no change [22, 23], and most surprisingly even reductions in amyloid compared to controls as well [24]. The basis for these conflicting PET findings may be due to methodological differences across studies. These include heterogeneity of depression with respect to age of first depressive episode, degree of response to treatment, magnitude of HPA axis and microglia activation accompanying each depressive episode as well as in the frequency of the e4 allele which have all been implicated in Aβ dynamics.

A reduction in plasma Aβ42/Aβ40 ratio has also been observed in the very early phases of AD and was found to be associated with future decline in cognition [25, 26]. Cross-sectional studies in depressed elderly adults have also reported decreased plasma Aβ42/Aβ40 ratios compared to age-matched controls [27]. However, to our knowledge, no longitudinal studies have evaluated plasma Aβ levels in subjects with late-life major depression (LLMD).

In this study, we tested the hypothesis that plasma Aβ levels differ between elderly individuals with LLMD and age-matched controls and that disease severity at baseline predicts long-term plasma Aβ status. We explored this hypothesis in a group of 48 cognitively intact depressed participants and 45 controls who were followed up longitudinally for 3 years. In addition, we examined whether plasma Aβ levels were accompanied by changes in cognitive performance and severity of depressive symptoms over time. Since brain derived neurotrophic factor (BDNF), a neurotrophin that plays an important role in cognition and depression [28, 29] is also known to regulate Aβ production and deposition [30, 31], we also examined the correlations between plasma BDNF and Aβ at Baseline.

## Methods

### Subjects and study design

The study was approved by the Nathan Kline Institute for Psychiatric Research and the New York University School of Medicine Institutional Review Boards. Participants were recruited via advertisements in local newspapers and flyers, or via the Memory Education and Research Initiative. Participants were prescreened over the phone during which a description of the study and participation requirements were described in detail before coming to the clinic for screening. Participants provided written informed consent prior to the study at the screening visit and were compensated up to $450 for their participation. One-hundred and thirty-three total participants were enrolled in the study. Thirty subjects did not complete the study: 3 died, 15 were lost to follow-up or did not return calls to schedule, 4 moved to a different location, and 8 withdrew from participation. Ten subjects completed all study visits but had unusable plasma samples and were excluded from the primary analysis. Of the 133 subjects entering the study, 93 completed all four study visits (baseline and three follow-up visits), had available plasma samples and were included in the primary analysis. Of these 93 subjects, 48 had a diagnosis at baseline of LLMD, and 45 were aged-matched controls.

In order to be enrolled in the study participants had to be 60 years, have a normal MRI scan (Fazeka score < 3), absence of unstable medical illness, and clinically normal lab values. To be enrolled into the LLMD group, participants had to meet the criteria for Major Depressive Disorder (MDD) based on the Structured Clinical Interview for the Diagnostic and Statistical Manual of Mental Disorders (DSM)-IV (SCID) conducted by a board-certified psychiatrist. Antidepressant use was allowed in the LLMD Group. To be enrolled in the control group, participants could not have a history of MDD or any other major psychiatric disorder. Additionally, all participants, regardless of LLMD or control group, had to be cognitively normal at screening. Normal cognition was defined as: Global Clinical Dementia Rating Scale score of 0 and a Mini-Mental State Exam score > 25.

### Study procedure

During the screening phase of the study, participants underwent testing at the Nathan Kline Institute or at the New York University Medical Center, over three successive visits, one week apart. During the first Screening Visit, participants provided informed consent, blood for *APOE* genotype was collected, and the Hamilton Depression Rating Scale (HAM-D) was administered. During the second Screening Visit, a general medical history was taken, the Mini-Mental State Examination (MMSE), the Geriatric Depression Scale (GDS) and Hamilton Anxiety Scale were also administered. During the third Screening Visit, participants received an MRI scan of the head and blood sample for routine laboratory tests was collected. After completing the screening phase and qualifying for the study, participants completed the Baseline Visit. During the Baseline Visit, participants underwent a full neuropsychological assessment, a psychiatric interview, a physical and neurological exam, and repeated mood scales (HAM-D, GDS, etc.). In general, the time between Screening and Baseline was approximately two weeks, but in some cases, there was a longer interval (i.e., up to a month). The three follow-up visits (Follow-up 1–3 Visits) took place in the same locations and were spaced approximately one-year apart. During the follow-up visits, participants completed the same comprehensive neuropsychological evaluation that they completed at the Baseline Visit. A clinical evaluation to assess for change in mood, a physical and neurological exam, the mood rating scales and routine laboratory tests were completed. Blood draws for Aβ40 and Aβ42 measurements were taken at Baseline and at each of the three follow-up visits.

### Measurements of depression status and cognitive performance

Depression status and severity were measured with the 21-item Hamilton Depression Rating Scale (HAM-D) which has a range of 0–55. Two measures of depression severity are captured from this measure, the HAMD-17, which sums the total of the first 17 items and the HAMD-21, which sums all items and has additional questions about depressive subtypes. The HAMD-17 is used to determine depression severity with a cut-off of 17 for moderate depression [32]. Global cognitive performance was assessed with the Mini-Mental State Examination (MMSE) which has a range of 0–30 and a score of 25 as education-adjusted cut-off for normality. Verbal learning and memory were measured, with the Buschke Selective Reminding Test Total score and 20-min Delayed Recall Score, which were selected from the larger neuropsychological test battery. Tests were administered at Baseline and at the 3 yearly follow-ups.

### Plasma Aβ measurement

Blood was collected in standard 10 ml EDTA tubes (BD Vacutainer). Immediately after blood draw, tubes were inverted gently 10–12 times for mixing and centrifuged at 3,000 rpm for 15 min at room temperature. Plasma aliquots were transferred to polypropylene low binding tubes and immediately stored at −80 °C. Plasma Aβ42 and Aβ40 concentrations were determined using biochemical the INNO-BIA plasma Aβ forms assay, format B (Fujirebio, Ghent, Belgium), a multiplex microsphere-based Luminex xMAP technique for simultaneous detection of Aβ X-42 and AβX–40 as previously described [33]. The plasma Aβ42/Aβ40 ratio was calculated by dividing the plasma Aβ42 by Aβ40 concentrations at each corresponding visit.

### Plasma BDNF measurement

BDNF protein levels in plasma samples were measured using an enzyme-linked immunosorbent assay (ELISA) method (BDNF Emax Immunoassay System, Promega, USA), according to the manufacturer’s instructions as described previously (see Pillai, Bruno [29]). Briefly, 96-well flat bottom immunoplates were incubated with an anti-BDNF monoclonal antibody at 4 °C overnight followed by incubation with standards and samples for 2 h at room temperature. Following washing with TBST wash buffer, plates were incubated for 2 h with Anti-Human BDNF polyclonal antibody. Subsequently, Anti-immunoglobulin Y-horse-radish peroxidase conjugate was added followed by the addition of TMB One solution to develop the color. The reaction was stopped by the addition of HCl 1 N and the absorbance was read at 450 nm on a microplate reader. BDNF concentrations were determined using the BDNF standard curve values (ranging from 7.8 to 500 pg/ml purified BDNF). All the samples were analyzed in duplicate in one session by an investigator blind to experimental set up.

### Statistical Analysis

Comparisons of variables measured longitudinally [Hamilton Depression Rating Scale Score (HAM-D), cognitive tests (MMSE, Buschke Selective Reminding Test Total and Delay Recall), Aβ42 and Aβ40 plasma levels and their ratio] between depressed and control subjects were performed with repeated-measures analysis of variance (RMANOVA). The potential effects of a number of variables at screening (MMSE score, age, education, diagnosis, HAM-D, antidepressant use, *APOE* genotype) on Aβ plasma levels at Year 3 were evaluated with stepwise linear regression and partial correlations. The main analyses were performed with all subjects completing the study (*n* = 93). Power calculations were determined before and after the study with the G*Power 3.1 software. For this sample size, the post-power analysis confirms that we had had a power (1-β) >0.85 in detecting significant (*α* = 0.05) differences equal or superior to 20% in plasma Aβ42, Aβ40 or Aβ42/Aβ40 between depressed and control groups. Sensitivity analyses were carried out excluding those subjects with abnormal MMSE score or MRI findings at Baseline (*n* = 6) and produced results very similar to those of the main analyses, so the reported findings include all subjects completing the study. Statistical analysis was run in SPSS v24.0. Alpha of *p* < 0.05 was used to indicate statistical significance. Figures were generated using Seaborn 0.10.0.

## Results

Table 1 summarizes the demographic and clinical characteristics. Antidepressant use is described in Supplemental Table S1. A total of 48 elderly LLMD (mean age at Baseline = 67.9 years) and 45 control elderly subjects (mean age at Baseline = 68.4 years) were followed over a 3-year period. Both the LLMD and the control groups differed only on the HAM-D total score (*p* < 0.001), with the LLMD group significantly more depressed than controls. Both groups were cognitively intact as measured by the MMSE at Baseline (LLMD: Mean score = 29.6; Controls: mean score = 29.6). We found no group differences in the proportion of individuals with factors known to influence Aβ dynamics, including in the number of ε4 or ε2 allele carriers and those with a family history of AD. Supplemental Table S2 shows demographic and clinical characteristics of the 40 subjects who were not included in the primary analysis. Their baseline characteristics were very similar to those of the 93 subjects included in the primary analysis. An analysis of cognition over time is found in the Supplemental Results. In summary, there were no groups differences in any of the cognitive indices at baseline and at the end of 3 years.

**Table 1:** Baseline demographic and clinical characteristics (standard deviations in parentheses) of the subjects completing the study.

Baseline scores	LLMD (*n* = 48)		Controls (*n* = 45)		*P*-value
Age (years)	67.9 (5.8)		68.4 (6.3)		0.740
Education (years)	16.5 (2.6)		16.5 (2.6)		0.856
MMSE Score	29.6 (0.9)		29.6 (0.9)		0.723
Hamilton depression rating scale score (HAM-D)	18.6(9.3)		1.5 (2.8)		<0.001
**Baseline frequencies**	**LLMD**		**Controls**		***P*-value**
	***N***	**%**	***N***	**%**	
Sex (Female)	24	50	24	53	0.748
Family history of Alzheimer’s disease	5	10	10	22	0.122
Apolipoprotein ε genotype (presence of at least one allele)					
ε2	10	21	12	27	0.508
ε3	45	94	43	96	0.700
ε4	15	31	7	16	0.075
Number of subjects taking antidepressants at Baseline	29	60	0	0	0.004
Number of subjects taking SSRIs at Baseline	17	35	0	0	<0.001

Continuous variables were compared with Student *t*-test, while frequencies were compared with Chi Square test.

### Severity of depression

**Fig. 1:**
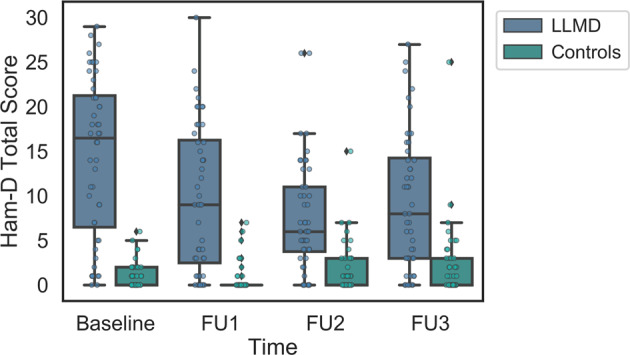
Boxplots of HAM-D Score. Boxplots with individual datapoints superimposed of total HAM-D score of the LLMD (blue) and control (green) groups and baseline and each yearly follow-up visits. *p < 0.001 between LLMD and controls.

Figure 1 shows mean total HAM-D scores of the LLMD and control groups at Baseline and at the 3 follow-up visits. Two-way RMANOVA indicates that mean HAM-D scores of the LLMD group were significantly different than those of the control group at all time points (*p* < 0.001). There was a significant interaction between the fixed factor time and diagnosis (*p* < 0.001) indicating that mean HAM-D scores in depressed patients significantly decreased over time compared to Baseline. Although, the LLMD groups’ depressive symptoms improved relative to Baseline, across the 3-year period the LLMD group remained significantly more depressed than the control group.

### Aβ plasma levels

Table 2 reports the means and standard deviations of plasma concentrations of Aβ42, Aβ40, and Aβ42/Aβ40 ratio across visits in the LLMD and controls groups.

**Table 2:** Means and standard deviations (SD) of plasma levels of Aβ40, Aβ42, and Aβ42/Aβ40 plasma ratio for cognitively intact participants with major depressive disorder (LLMD) and controls at Baseline and Follow-Up Visits 1, 2 and 3.

Plasma Aβ	Time	LLMD	Controls	*t*-test
		(*n* = 48)	(*n* = 45)	(*p*-value)
**Aβ42 (pg/mL)**	Baseline	17.9 (6.6)	17.9 (5.5)	0.942
	FU1	19.0 (6.8)	21.5 (5.6)	0.052
	FU2	19.4 (6.4)	21.2 (6.1)	0.170
	FU3	19.8 (6.8)	20.7 (5.4)	0.497
**Aβ40 (pg/mL)**	Baseline	143.3 (39.6)	122.6 (39.2)	0.013*
	FU1	158.8 (41.4)	148.3 (40.9)	0.222
	FU2	151.7 (31.7)	148.1 (42.4)	0.645
	FU3	155.7 (40.1)	145.3 (39.8)	0.213
**Aβ42/Aβ40**	Baseline	0.13 (0.1)	0.15 (0.5)	0.005*
	FU1	0.12 (0.4)	0.15 (0.5)	0.001*
	FU2	0.13 (0.4)	0.15 (0.5)	0.031*
	FU3	0.13 (0.2)	0.15 (0.4)	0.082

Univariate comparisons using independent samples *t*-test with the corresponding *p*-value for each timepoint are listed. *Significant difference at *p* < 0.05 level between LLMD and controls.

**Fig. 2:**
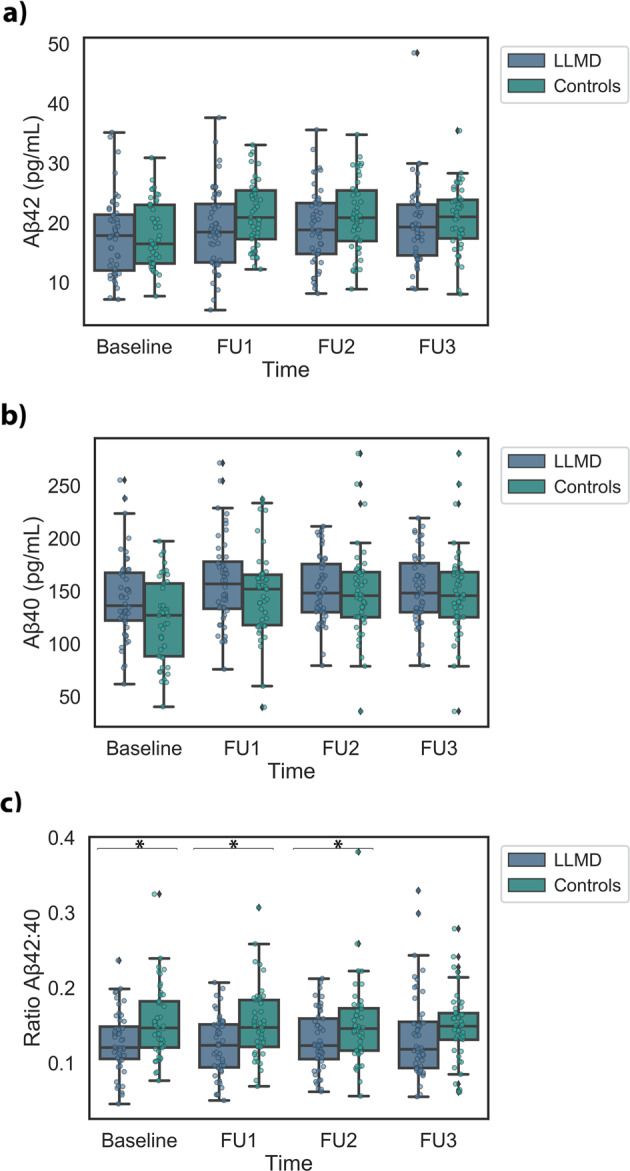
Boxplots of Plasma Aβ Variables. Boxplots with individual datapoints superimposed of plasma Aβ42 (panel **a**), Aβ40 (panel **b**) and Aβ42/Aβ40 ratio (panel **c**) of the LLMD and controls groups at Baseline and at the follow-up visits (FU). *p < 0.01 between LLMD and controls.

Although small changes were observed in Aβ42 and Aβ40 over time (Table 3), the variations were similar in depressed and control groups and no significant variations of the plasma concentrations were observed for the two peptides over time. Figure 2 shows boxplots of plasma Aβ42, Aβ40 and Aβ42/Aβ40 ratio of the LLMD and controls groups at Baseline and at the follow-up visits. Results of two-way mixed RMANOVA did not reveal significant differences between LLMD and control groups in either Aβ42 or Aβ40 plasma levels. However, the mean Aβ42/Aβ40 plasma ratios of the LLMD group were significantly lower than those of the control group at all time points (*p* = 0.005) (Fig. 2c). There was no significant interaction between time and diagnosis indicating that the temporal trends of the mean Aβ42/Aβ40 ratio of the two groups were parallel. We did not find significant differences in mean Aβ42/Aβ40 plasma ratio at Baseline (*p* = 0.93) between LLMD patients taking antidepressants and those not treated (mean = 0.128 (SD = 0.046). and 0.129 (SD = 0.034), respectively).

**Table 3:** Repeated Measure ANOVA F-Test results for testing the interaction between time and groups (depressed and controls) for HAM-D, Cognitive Tests, Aβ40, Aβ42, and Aβ42/Aβ40 ratio.

Outcome variable	F test (within–subject)		*P*-value	Effect size (*η*^2^)
	df **	F statistic		
**HAM-D**	2.64, 240.38	15.18	*p* < 0.001*	0.14
**MMSE**	2.43, 221.26	0.53	0.627	0.006
**Total recall**	2.87, 261.11	3.63	0.015*	0.04
**Delay recall**	2.93, 266.71	2.47	0.064	0.03
**Aβ40**	2.85, 259.26	1.51	0.214	0.02
**Aβ42**	2.79, 254.01	1.69	0.174	0.02
**Aβ42/Aβ40**	2.63, 239.09	1.66	0.184	0.02
**Outcome variable**	**F test (between–subject)**		***P*-value**	**Effect size** **(*η***^2^)
	df	F statistic		
**Aβ42/Aβ40**	1.91	8.205	0.005*	0.08

*Significant interaction of time and diagnosis. **degrees of freedom adjusted for violations of Mauchly’s test using the Huynh-Fedlt correction.

### Association of depressive symptoms and future plasma Aβ levels at follow up

We used a stepwise linear regression to model the associations of depressive symptoms at screening and the Aβ42/Aβ40 plasma ratios at the different visits (Table 3). The predictors included MMSE score, age, education, HAM-D, *APOE* status and antidepressant use at screening. Since there was a significant reduction in the HAM-D total score from the screening to the baseline visit we tested both the Screening and the Baseline HAM-D score in predicting the Aβ42/Aβ40 plasma ratio. We found that HAM-D scores collected at screening significantly predicted Aβ42/Aβ40 ratio at year 1 (*β* = −0.33, *p* = 0.001) and year 3 (*β* = −0.24, *p* = 0.018). Similarly, the HAM-D score measured at baseline also significantly predicted Aβ42/Aβ40 ratio at year 1 (*p* = .003) and year 3 (*p* = 0.040). We also examined possible differences in subjects with early- (<60 years, *n* = 29) vs late- (≥60 years, *n* = 19) onset of LLMD. Neither age of first depressive episode (mean ± SD = 53.5 ± 12.0 years) nor first onset at age 60 or older was significantly correlated with the plasma Aβ42/Aβ40 ratio, in LLMD, at any timepoint.

### Relationship between plasma Aβ and BDNF

The relationship between plasma Aβ and BDNF was explored using Pearson correlations. There were no significant correlations between plasma Aβ42, Aβ40 or Aβ42/Aβ40 ratio and BDNF for the whole group. There were also no significant correlations for LLMD or controls, or for males and females, when run separately.

## Discussion

We found that the plasma Aβ42/Aβ40 ratio was consistently lower in depressed subjects compared to controls over a period of 3 years. This finding is consistent with previous cross-sectional studies indicating a reduced plasma Aβ42/Aβ40 ratio in older adults with late-life depression [27]. Since no significant differences were observed in cognitive performance between depressed and control groups, or as a function of plasma Aβ levels, it is unlikely that greater cognitive decline contributed to the lower plasma Aβ42/Aβ40 ratio in the LLMD group. Our study also identified an association of depressive symptoms at baseline and the plasma Aβ42/Aβ40 ratios in subsequent visits. This finding supports the hypothesis that depressive symptoms may drive plasma Aβ dynamics, which should be explored in further studies.

The lower plasma Aβ42/Aβ40 ratio we observed in elderly depressed subjects was associated with higher plasma Aβ40 with no significant group difference in plasma Aβ42 levels. Thus, future studies should determine if this peptide alone contributes to the significantly lower Aβ42/Aβ40 ratio found in LLMD. It also is possible that the plasma Aβ42/Aβ40 ratio is a more sensitive biomarker of Aβ dynamics since it represents the equilibrium of the two, rather than a single peptide. This conclusion agrees with the generally accepted notion that Aβ40 serves to control for inter-individual differences in total Aβ production, as well as possible pre-analytical confounders [34]. Although the precise origin of circulating blood Aβ is not known [35], numerous studies in AD support that a reduction in the plasma Aβ42/Aβ40 ratio is associated with brain amyloidosis [36, 37]. Therefore, future studies, using amyloid PET, should validate whether the decreased plasma Aβ42/Aβ40 ratio which we observed in elderly depressives reflects increased brain amyloid burden or changes in soluble Aβ levels, especially circulating Aβ40, which may be independent of brain amyloid load.

Previous studies have examined brain and CSF amyloid indices in elderly depressives; however, the number of studies is limited and the results conflicting. In a subgroup of the cohort described in the current report, mean CSF Aβ42 levels at baseline were significantly lower in depressed subjects compared to controls but then increased over time and were no longer significantly different from controls after 3 years [38]. The increase in CSF Aβ42 levels in depressed subjects coincided with improvement in depressive symptoms. These findings were consistent with state-dependent effects of depressive symptoms on CSF Aβ42 rather than with the prevailing opinion that this disorder might reflect prodromal AD. This interpretation is also supported by our current findings that no notable differences in cognitive ability were observed across groups, or as a function of Aβ levels, although we cannot rule out that these differences might yet emerge later. Furthermore, the plasma Aβ42/Aβ40 ratio at 3 years was predicted by baseline severity of depressive symptoms, and not by cognitive indices.

Statistical analyses indicated that group differences in plasma Aβ42/Aβ40 ratio that we observed cannot be ascribed to differences in the frequency of the ε4 allele or to a family history of AD. In addition, individuals with a first episode of depression earlier in life (EOMD) showed a reduction in the plasma Aβ42/Aβ40 ratio that was comparable to that observed in individuals with late onset major depression (LOMD). This suggests that the reduction found in plasma Aβ42/Aβ40 levels in EOMD is unlikely to reflect preclinical AD and is consistent with epidemiological data that depression early in life may be a risk factor for AD [39]. Furthermore, these findings raise the possibility that in cognitively intact depressed elderly, factors intrinsic to depression such as increased severity of symptoms might influence Aβ metabolism independently of preclinical or prodromal AD, or alternatively that brain Aβ deposition that does not lead to overt cognitive symptoms (i.e., in the absence of neurofibrillary tangles or neuronal death) promotes depressive symptoms in the elderly. Several lines of evidence from the preclinical and clinical literature have presented compelling evidence that the applications of various types of stress, sleep deprivation, administration or elevations in corticosterone or corticotropin-releasing hormone, which have all been implicated in depression, can result in significant elevation in soluble and aggregated forms of Aβ [40, 41] as well as elevation in total and phosphorylated tau [42]. These effects may be in part mediated by brain-region-specific increases in neuronal activity which has been reported in depression [43]. Additionally, a subtle loss of hippocampal integrity associated with depression, might result in a reduction of inhibitory input to the hypothalamic-pituitary-adrenal axis and cortisol elevations which have been implicated in altered Aβ metabolism [44].

The reduced plasma Aβ42/Aβ40 ratio which we observed in LLMD subjects may involve both central and peripheral mechanisms. A decrease in plasma Aβ42/Aβ40 ratio is observed also in both AD subjects and in those with early or prodromal AD stages [45, 46] and has been associated to accumulation of Aβ42 in brain deposits with consequent sequestration of the Aβ42 peptide from CSF and plasma. A similar mechanism must be excluded in LLMD because it has been demonstrated that the longitudinal pattern of CSF Aβ42 concentrations does not depend on cognitive status but reflects depression severity with lower levels of CSF Aβ42 associated with more severe depressive symptoms [38]. Increased platelet activation associated with depression [47] may have also influenced plasma Aβ42/Aβ40 ratio levels since platelets are known to produce Aβ, particularly Aβ40, regardless of the increase in brain Aβ deposition [48]. The increase in circulating levels of Aβ40 has also been implicated in the increase in Aβ plaques formation through a number of mechanisms, including NF-kB-dependent endothelial cell activation, neuroinflammation, increase in APP metabolism and Aβ production [49, 50]. Therefore, future studies should determine whether the increased platelet activation associated with depression causes an increase in circulating Aβ40 levels which in turn increases brain Aβ deposition and consequently the risk of AD onset.

Although previous studies have shown an association between plasma BDNF levels and amyloid burden, our findings did not show significant association between BDNF and Aβ indices. The lack of correlation between plasma BDNF and Aβ42, or Aβ40 in the plasma samples in our study could be due the differences in the methodology used, tissue samples examined (plasma vs. brain) and/or the subject population (AD vs late-life depression). In addition, the ELISA kit used in our study does not differentiate proBDNF and mature BDNF levels. ProBDNF is known to enhance Aβ levels and accelerate its deposition in the brain [51]. Future studies using isoform specific BDNF antibodies are warranted to examine the relative roles of mature and proBDNF on Aβ burden.

The main limitation of this study is the small sample size. Additionally, the results of this study were focused on plasma indices. Although we collected CSF Aβ measures, it was only in a small subset. Therefore, we were not able to formally compare the plasma results with the corresponding CSF measures as there was an insufficient number of subjects with both plasma and CSF Aβ determinations. In addition, our study did not examine the relationship between lower plasma Aβ42/Aβ40 ratio and indices of the HPA axis and microglial activation as well as whole brain and region-specific PET amyloid binding [52].

Future studies of LLMD should consider collecting CSF, plasma, and brain indices to further elucidate the relationship between these measures. Additionally, future studies should explore plasma Aβ indices using mass spectrometry or ultrasensitive immunoassays (Simoa) with higher sensitivity and specificity for reliably measuring Aβ and tau levels in blood [53] in this population to determine their relationship to depression diagnosis and to its aforementioned clinical features. If confirmed, more effective treatment of depressive symptoms, insomnia, and platelet activation in patients with depression may lead to a lowering of AD biomarkers and the associated risk of AD. Additionally, an important clinical question is whether we can delay the onset of AD in some individuals by treating their depressive symptoms.

In conclusion, our study demonstrates that the association of depression and plasma Aβ status is robust. Future studies should determine if successful treatment of depressive symptoms in cognitively normal depressed subjects will impact the longitudinal plasma Aβ42/Αβ40 ratio and the potential long-term AD risk.

## Supplementary information

Supplemental Results

## Data Availability

The data generated during the current study are not publicly available because the approved IRB protocol does not include a provision for deposit in a public repository. The data may be made available from the corresponding author on reasonable request.

## References

[1] Green RC, Cupples LA, Kurz A, Auerbach S, Go R, Sadovnick D (2003). Depression as a risk factor for Alzheimer disease: the MIRAGE study. Arch Neurol.

[2] Byers AL, Yaffe K (2011). Depression and risk of developing dementia. Nat Rev Neurol.

[3] Dal Forno G, Palermo MT, Donohue JE, Karagiozis H, Zonderman AB, Kawas CH (2005). Depressive symptoms, sex, and risk for Alzheimer’s disease. Ann Neurol.

[4] Steffens DC, Plassman BL, Helms MJ, Welsh-Bohmer KA, Saunders AM, Breitner JC (1997). A twin study of late-onset depression and apolipoprotein E ε4 as risk factors for Alzheimer’s disease. Biol Psychiatry.

[5] Jorm AF (2001). History of depression as a risk factor for dementia: an updated review. Aust NZ J Psychiatry.

[6] Ownby RL, Crocco E, Acevedo A, John V, Loewenstein D (2006). Depression and risk for Alzheimer disease: systematic review, meta-analysis, and metaregression analysis. Arch Gen Psychiatry.

[7] Wilson RS, Barnes L, De Leon CM, Aggarwal N, Schneider J, Bach J (2002). Depressive symptoms, cognitive decline, and risk of AD in older persons. Neurology.

[8] Rapp MA, Schnaider-Beeri M, Grossman HT, Sano M, Perl DP, Purohit DP (2006). Increased hippocampal plaques and tangles in patients with Alzheimer disease with a lifetime history of major depression. Arch Gen Psychiatry.

[9] Rapp MA, Schnaider-Beeri M, Purohit DP, Perl DP, Haroutunian V, Sano M (2008). Increased neurofibrillary tangles in patients with Alzheimer disease with comorbid depression. Am J Geriatr Psychiatry.

[10] Becker JT, Chang Y-F, Lopez OL, Dew MA, Sweet RA, Barnes D (2009). Depressed mood is not a risk factor for incident dementia in a community-based cohort. Am J Geriatr Psychiatry.

[11] Rozzini L, Chilovi BV, Trabucchi M, Padovani A (2005). Depression is unrelated to conversion to dementia in patients with mild cognitive impairment. Arch Neurol.

[12] Ganguli M, Du Y, Dodge HH, Ratcliff GG, Chang C-CH (2006). Depressive symptoms and cognitive decline in late life: a prospective epidemiological study. Arch Gen Psychiatry.

[13] Pomara N, Bruno D, Sarreal AS, Hernando RT, Nierenberg J, Petkova E (2012). Lower CSF amyloid beta peptides and higher F2-isoprostanes in cognitively intact elderly individuals with major depressive disorder. Am J Psychiatry.

[14] Wu K-Y, Hsiao T, Chen C-S, Chen C-H, Hsieh C-J, Wai Y-Y (2014). Increased brain amyloid deposition in patients with a lifetime history of major depression: evidenced on 18 F-florbetapir (AV-45/Amyvid) positron emission tomography. Eur J Nucl Med Mol Imaging.

[15] Yasuno F, Kazui H, Morita N, Kajimoto K, Ihara M, Taguchi A (2016). High amyloid‐β deposition related to depressive symptoms in older individuals with normal cognition: a pilot study. Int J Geriatr Psychiatry.

[16] Ovod V, Ramsey KN, Mawuenyega KG, Bollinger JG, Hicks T, Schneider T (2017). Amyloid β concentrations and stable isotope labeling kinetics of human plasma specific to central nervous system amyloidosis. Alzheimer’s Dement.

[17] Nakamura A, Kaneko N, Villemagne VL, Kato T, Doecke J, Doré V (2018). High performance plasma amyloid-β biomarkers for Alzheimer’s disease. Nature.

[18] Li P, Hsiao I-T, Liu C-Y, Chen C-H, Huang S-Y, Yen T-C (2017). Beta-amyloid deposition in patients with major depressive disorder with differing levels of treatment resistance: a pilot study. EJNMMI Res.

[19] Tateno A, Sakayori T, Higuchi M, Suhara T, Ishihara K, Kumita S (2015). Amyloid imaging with [18F] florbetapir in geriatric depression: early‐onset versus late‐onset. Int J Geriatr Psychiatry.

[20] Kumar A, Kepe V, Barrio JR, Siddarth P, Manoukian V, Elderkin-Thompson V (2011). Protein binding in patients with late-life depression. Arch Gen Psychiatry.

[21] Smith GS, Kuwabara H, Nandi A, Gould NF, Nassery N, Savonenko A (2021). Molecular imaging of beta-amyloid deposition in late-life depression. Neurobiol Aging.

[22] Takamiya A, Vande Casteele T, Koole M, De Winter F-L, Bouckaert F, Van den Stock J (2021). Lower regional gray matter volume in the absence of higher cortical amyloid burden in late-life depression. Sci Rep..

[23] Madsen K, Hasselbalch BJ, Frederiksen KS, Haahr ME, Gade A, Law I (2012). Lack of association between prior depressive episodes and cerebral [11C] PiB binding. Neurobiol Aging.

[24] Mackin RS, Insel PS, Landau S, Bickford D, Morin R, Rhodes E (2021). Late-life depression is associated with reduced cortical amyloid burden: findings from the Alzheimer’s disease neuroimaging initiative depression project. Biol Psychiatry.

[25] Schindler SE, Bollinger JG, Ovod V, Mawuenyega KG, Li Y, Gordon BA (2019). High-precision plasma β-amyloid 42/40 predicts current and future brain amyloidosis. Neurology.

[26] Kim HJ, Park KW, Kim TE, Im JY, Shin HS, Kim S (2015). Elevation of the plasma Aβ 40/Aβ 42 ratio as a diagnostic marker of sporadic early-onset Alzheimer’s disease. J Alzheimer’s Dis.

[27] do Nascimento KKF, Silva KP, Malloy-Diniz LF, Butters MA, Diniz BS (2015). Plasma and cerebrospinal fluid amyloid-β levels in late-life depression: a systematic review and meta-analysis. J Psychiatr Res.

[28] Autry AE, Monteggia LM (2012). Brain-derived neurotrophic factor and neuropsychiatric disorders. Pharmacol Rev.

[29] Pillai A, Bruno D, Sarreal AS, Hernando RT, Saint-Louis LA, Nierenberg J (2012). Plasma BDNF levels vary in relation to body weight in females. PloS One.

[30] Hwang KS, Lazaris AS, Eastman JA, Teng E, Thompson PM, Gylys KH (2015). Plasma BDNF levels associate with Pittsburgh compound B binding in the brain. Alzheimer’s Dement.

[31] Nigam SM, Xu S, Kritikou JS, Marosi K, Brodin L, Mattson MP (2017). Exercise and BDNF reduce Aβ production by enhancing α‐secretase processing of APP. J Neurochemistry.

[32] Zimmerman M, Martinez JH, Young D, Chelminski I, Dalrymple K (2013). Severity classification on the Hamilton depression rating scale. J Affect Disord.

[33] Hansson O, Zetterberg H, Vanmechelen E, Vanderstichele H, Andreasson U, Londos E (2010). Evaluation of plasma Aβ40 and Aβ42 as predictors of conversion to Alzheimer’s disease in patients with mild cognitive impairment. Neurobiol Aging.

[34] Blennow K, Zetterberg H (2018). Biomarkers for Alzheimer’s disease: current status and prospects for the future. J Intern Med.

[35] Uddin MS, Kabir MT, Tewari D, Al Mamun A, Mathew B, Aleya L et al. Revisiting the role of brain and peripheral Aβ in the pathogenesis of Alzheimer’s disease. J. Neurol. Sci. 2020;416:116974. 10.1016/j.jns.2020.116974 32559516

[36] Li Y, Schindler SE, Bollinger JG, Ovod V, Mawuenyega KG, Weiner MW (2022). Validation of plasma amyloid-β 42/40 for detecting Alzheimer disease amyloid plaques. Neurology.

[37] Hu Y, Kirmess KM, Meyer MR, Rabinovici GD, Gatsonis C, Siegel BA (2022). Assessment of a plasma amyloid probability score to estimate amyloid positron emission tomography findings among adults with cognitive impairment. JAMA Netw Open.

[38] Pomara N, Bruno D, Osorio RS, Reichert C, Nierenberg J, Sarreal AS (2016). State-dependent alterations in CSF Abeta42 levels in cognitively intact elderly with late life major depression. Neuroreport.

[39] Geerlings M, den Heijer T, Koudstaal P, Hofman A, Breteler M (2008). History of depression, depressive symptoms, and medial temporal lobe atrophy and the risk of Alzheimer disease. Neurology.

[40] Dong H, Yuede C, Yoo H-S, Martin M, Deal C, Mace A (2008). Corticosterone and related receptor expression are associated with increased β-amyloid plaques in isolated Tg2576 mice. Neuroscience.

[41] Dong H, Murphy KM, Meng L, Montalvo-Ortiz J, Zeng Z, Kolber BJ (2012). Corticotrophin releasing factor accelerates neuropathology and cognitive decline in a mouse model of Alzheimer’s disease. J Alzheimer’s Dis.

[42] Sotiropoulos I, Catania C, Pinto LG, Silva R, Pollerberg GE, Takashima A (2011). Stress acts cumulatively to precipitate Alzheimer’s disease-like tau pathology and cognitive deficits. J Neurosci.

[43] Backes H, Dietsche B, Nagels A, Stratmann M, Konrad C, Kircher T (2014). Increased neural activity during overt and continuous semantic verbal fluency in major depression: mainly a failure to deactivate. Eur Arch Psychiatry Clin Neurosci.

[44] Du X, Pang TY (2015). Is dysregulation of the HPA-axis a core pathophysiology mediating co-morbid depression in neurodegenerative diseases?. Front Psychiatry.

[45] Verberk IM, Slot RE, Verfaillie SC, Heijst H, Prins ND, van Berckel BN (2018). Plasma amyloid as prescreener for the earliest Alzheimer pathological changes. Ann Neurol.

[46] Janelidze S, Stomrud E, Palmqvist S, Zetterberg H, Van Westen D, Jeromin A (2016). Plasma β-amyloid in Alzheimer’s disease and vascular disease. Sci Rep..

[47] Musselman DL, Marzec UM, Manatunga A, Penna S, Reemsnyder A, Knight BT (2000). Platelet reactivity in depressed patients treated with paroxetine: preliminary findings. Arch Gen Psychiatry.

[48] Chen M, Inestrosa NC, Ross GS, Fernandez HL (1995). Platelets are the primary source of amyloid β-peptide in human blood. Biochemical biophysical Res Commun.

[49] Humpel C (2017). Platelets: their potential contribution to the generation of beta-amyloid plaques in Alzheimer’s disease. Curr Neurovascular Res.

[50] Sagare AP, Bell RD, Zlokovic BV (2013). Neurovascular defects and faulty amyloid-β vascular clearance in Alzheimer’s disease. J Alzheimer’s Dis.

[51] Chen J, Zhang T, Jiao S, Zhou X, Zhong J, Wang Y (2017). ProBDNF accelerates brain amyloid-β deposition and learning and memory impairment in APPswePS1dE9 transgenic mice. J Alzheimer’s Dis.

[52] Pagni G, Tagliarini C, Carbone MG, Imbimbo BP, Marazziti D, Pomara N. Different sides of depression in the elderly: an in-depth view on the role of Aβ peptides. Curr Med Chem. 2022. 10.2174/0929867328666210921164816. 10.2174/0929867328666210921164816 34547994

[53] Li D, Mielke MM (2019). An update on blood-based markers of Alzheimer’s disease using the SiMoA platform. Neurol Ther.

