# Supplementary material for: Plasma Amyloid-β dynamics in late-life major depression: a longitudinal study
Source: Transl Psychiatry. 2022 Jul 28;12:301. doi: 10.1038/s41398-022-02077-8 (PMC9334636; doi:10.1038/s41398-022-02077-8)
Supplement: Supplementary file 1 — Supplemental Results [file 41398_2022_2077_MOESM1_ESM.docx]

**Plasma Amyloid-β Dynamics in late-life major depression: A longitudinal study**

**Running Title:** Plasma Amyloid-β Dynamics in LLMD

Nunzio Pomara, MD 1,2, Davide Bruno, PhD 3, Chelsea Reichert Plaska, PhD 1,4, Jaime Ramos-Cejudo, PhD 4, Ricardo S Osorio, MD 1,4, Anilkumar Pillai, PhD, 5, 6, 7 Bruno P. Imbimbo, PhD 8, Henrik Zetterberg, MD, PhD 9, 10, 11, 12, 13 and Kaj Blennow, MD, PhD 11, 12

(1) Nathan Kline Institute, Orangeburg, NY, USA, (2) Department of Psychiatry and Pathology, New York University-Grossman School of Medicine, New York, NY, USA, (3) School of Psychology, Liverpool John Moores University, Liverpool, United Kingdom, (4) Department of Psychiatry, New York University-Grossman School of Medicine, New York, NY, USA, (5) Pathophysiology of Neuropsychiatric Disorders Program, Faillace Department of Psychiatry and Behavioral Sciences, McGovern Medical School, The University of Texas Health Science Center at Houston (UTHealth), Houston, TX, USA, (6) Research and Development, Charlie Norwood VA Medical Center, Augusta, GA, USA, (7) Department of Psychiatry and Health Behavior, Medical College of Georgia, Augusta University, Augusta, GA, USA. (8) Research & Development, Chiesi Farmaceutici, Parma, Italy, (9) Department of Neurodegenerative Disease, UCL Institute of Neurology, London, United Kingdom, (10) UK Dementia Research Institute at UCL, London, United Kingdom, (11) Department of Psychiatry and Neurochemistry, Institute of Neuroscience and Physiology, the Sahlgrenska Academy at the University of Gothenburg, Mölndal, Sweden, (12) Clinical Neurochemistry Laboratory, Sahlgrenska University Hospital, Mölndal, Sweden (13) Hong Kong Center for Neurodegenerative Diseases, Clear Water Bay, Hong Kong, China

Corresponding Author: Nunzio Pomara, MD, Nathan Kline Institute for Psychiatric Research, Orangeburg, NY, 10962, USA

**Supplemental Results**

Supplemental Table 2 shows demographic and clinical characteristics of the 40 subjects that were not included in the primary analysis. Their baseline characteristics were very similar to those of the 93 subjects included in the primary analysis.

***Cognitive Performance***

Supplemental Table 3 shows mean values of cognitive tested at Baseline and at subsequent yearly follow-ups for the depressed and control groups. There was an interaction between time and diagnosis, for Total Recall only (p = 0.015) and a marginally significant interaction for Delay Recall (p = 0.064). Total Recall declined slight for the LLMD group between the Year 2 and Year 3 visits. However, there was no significant differences between LLMD and controls at these timepoints. Importantly, the two groups did not differ on the cognitive measures, including MMSE, at the other timepoints (see Supplemental Table 3).

**Supplemental Table 1**. List of antidepressants taken during the 3-year study. Antidepressants are listed as the generic name and classified as conventional antidepressants (i.e., SSRI, SNRI, etc.) or non-conventional antidepressants (i.e., antipsychotics, etc.).

| **Medication** | **Conventional antidepressant** | **Non-Conventional antidepressant** |
| --- | --- | --- |
| Adderall |  | X |
| Aripiprazole |  | X |
| Bupropion | X |  |
| Citalopram | X |  |
| Clonazepam |  | X |
| Desvenlafaxine | X |  |
| Duloxetine | X |  |
| Escitalopram | X |  |
| Fluoxetine | X |  |
| Lamotrigine |  | X |
| Mirtazapine | X |  |
| Olanzapine |  | X |
| Oxcarbazepine |  | X |
| Paroxetine | X |  |
| Quetiapine |  | X |
| Sertraline | X |  |
| Sodium Valproate |  | X |
| Trazodone |  | X |
| Venlafaxine | X |  |

**Supplemental Table 2**. Baseline demographic and clinical characteristics (standard deviations in parentheses) of the subjects (n = 40) that were not included in the primary analysis.

| **Baseline Scores** | **LLMD**  **(n=26)** | **Controls**  **(n=14)** |
| --- | --- | --- |
| Age (years) | 65.1 (4.7) | 68.29 (6.4) |
| Education (years) | 14.65 (2.4) | 16.4 (2.3) |
| MMSE Score | 29.42 (0.9) | 29.29 (1.1) |
| Hamilton Depression Rating Scale Score (Ham-D) | 18.62 (10.3) | 1.57 (3.2) |

| **Supplemental Table 3.** Mean and SD of main cognitive scores at Baseline and each yearly follow-up visits of the LLMD and control groups. There were no significant differences between LLMD and control groups for any of the variables at any time points.   \|  \| **Time** \| **LLMD (n = 48)** \| **Controls**  **(n=45)** \| **t-test**  **(p-value)** \| \| --- \| --- \| --- \| --- \| --- \| \| **MMSE** \| Baseline \| 29.6 (0.9) \| 29.6 (0.9) \| 0.723 \| \| FU1 \| 29.5 (0.8) \| 29.6 (0.7) \| 0.703 \| \| FU2 \| 29.7 (0.7) \| 29.5 (0.5) \| 0.225 \| \| FU3 \| 29.5 (1.0) \| 29.4 (1.2) \| 0.883 \| \| **Total Recall** \| Baseline \| 63.8 (14.2) \| 64.8 (15.6) \| 0.746 \| \| FU1 \| 66.5 (16.7) \| 63.0 (17.5) \| 0.326 \| \| FU2 \| 64.8 (17.1) \| 66.4 (16.1) \| 0.634 \| \| FU3 \| 60.6 (17.2) \| 65.9 (16.2) \| 0.131 \| \| **Delayed Recall** \| Baseline \| 8.4 (3.2) \| 8.7 (3.2) \| 0.707 \| \| FU1 \| 9.3 (3.8) \| 8.7 (3.7) \| 0.460 \| \| FU2 \| 8.4 (4.3) \| 9.2 (3.5) \| 0.331 \| \| FU3 \| 8.0 (4.0) \| 8.6 (3.9) \| 0.438 \| |
| --- | --- | --- | --- | --- | --- | --- | --- | --- | --- | --- | --- | --- | --- | --- | --- | --- | --- | --- | --- | --- | --- | --- | --- | --- | --- | --- | --- | --- | --- | --- | --- | --- | --- | --- | --- | --- | --- | --- | --- | --- | --- | --- | --- | --- | --- | --- | --- | --- | --- | --- | --- | --- | --- | --- | --- | --- |
